# Cancer seeding contributes to intestinal anastomotic dehiscence

**DOI:** 10.1186/1477-7819-11-302

**Published:** 2013-11-25

**Authors:** Marek Stanczyk, Waldemar L Olszewski, Magdalena Gewartowska, Marek Maruszynski

**Affiliations:** 1 Deptartment of Human Epigenetics, Mossakowski Medical Research Centre, Polish Academy of Science, Warsaw, Poland; 2 Department of General, Oncologic and Vascular Surgery, Military Institute of Medicine, Warsaw, Poland; 3 Department of General, Oncologic and Trauma Surgery, Wolski Hospital, Warsaw, Poland

## Abstract

**Background:**

Surgical wounds in cancer patients have a relatively high dehiscence rate. Although colon cancer resections are performed so as to include macroscopically non-involved tissues, some cancer cells can be present in the line of transection. The local healing process may facilitate proliferation of these localized cancer cells and the high cytokine concentration within the healing wound may also attract cancer cells from distant sites to migrate into the wound area. The growing tumor cells may then stretch the wound, hampering its contraction process.

**Methods:**

The aim of the study was to monitor and compare, using immunohistochemical methods, the healing process of intestinal anastomosis in both normal rats and in rats with disseminated cancer (the CC531 colon cancer model).

**Results:**

There was a significantly higher rate of anastomotic dehiscence in the group of rats with disseminated cancer, than in the group of normal rats. There were no significant differences between the two groups in the levels of mononuclear wound infiltration or of formation of connective tissue or new vessels. All anastomotic wounds in animals with disseminated cancer had abundant infiltrates of both migrating and proliferating cancer cells.

**Conclusions:**

We confirmed that the environment of a healing wound attracts cancer cells. Migration of cancer cells to the wound and centrifugal cancer proliferation may adversely affect the healing process and cause wound disruption.

## Background

The normal wound-healing process can be divided into three stages: 1) inflammatory, 2) proliferative, and 3) repair and remodeling. The inflammatory stage is initiated by blood coagulation and platelet degranulation. In response to released chemotactic factors, monocytes enter the wound and mature into wound macrophages. Wound macrophages phagocytose wound debris, and in conjunction with infiltrating lymphocytes, release growth factors, which induce migration and proliferation of fibroblasts, epithelial cells, and endothelial cells during the proliferative phase of healing. At the end of the proliferative phase, fibroblasts produce collagen, elastin, proteoglycans, and other extracellular matrix (ECM) components, resulting in scar tissue formation [1,2]. Remodeling and repair of scar tissue is controlled by action of metalloproteinases secreted by fibroblasts and downregulated by production of tissue inhibitor of matrix metalloproteinases (TIMPs). A number of reports confirmed observations that inflammation may be an important cofactor of tumorgenesis in sites of chronic irritation, persistent infection, and previously wounded tissue [3,4]. Macrophages also play a role in tumor growth in conjunction with lymphocytes, by synthesizing and secreting epidermal growth factor (EGF), basic fibroblast growth factor, and transforming growth factor (TGF) α and β along with other chemokines released during wound healing and inflammation, including tumor necrosis factor-α, interleukin 6, platelet-derived growth factor (PDGF) and vascular endothelial growth factor (VEGF) [5-9]. Tumor growth results in disruption of the normal tissue architecture, and induces a wound-healing response similar to that found in the normal healing wound. Because of these similarities, tumors are often described as ‘wounds that do not heal’ [10,11]. There is clinical evidence that complicated wound healing and local or systemic inflammation worsens prognosis in patients undergoing oncologic treatment. The delayed wound healing is associated with increased rate of systemic but not local recurrence after breast cancer surgery [12]. The anastomotic leakage after colorectal cancer surgery might enhance the incidence of local recurrence, and contribute to worse prognosis [13-17].

Surgical wounds in cancer patients have a higher dehiscence rate. Excision of tumor tissue is aimed at removal of the bulk of the tumor mass. However, even if tissue is transected at a large distance from the tumor edge, it is possible that individual tumor cells may be present in the presumed non-cancerous tissues. Presence of cancer at the anastomotic margin contributes to anastomotic leakage and ‘suture-line’ recurrence. This situation is rarely encountered in modern-day treatment, as the principles of negative proximal and distal margins are well appreciated, and the use of frozen section control of resection margins (if close or doubtful) is standard practice.

The local healing process may facilitate cancer cell proliferation within the wound, and the high levels of cytokines produced during the healing process may attract cancer cells from distant tissues to migrate to and proliferate within the wound [18]. These residual or newly attracted tumor cells then become involved in the wound-healing process [19].

The question arises as to how tumor cells react in all three stages of normal tissue healing compared with the local parenchyma and mesenchymal cells adjacent to the wound. Tumor cells proliferate with a net mass increase, whereas the neighboring normal tissue undergoes retraction and scar formation. It is therefore possible that the growing tumor cells will stretch the wound, hampering the process of wound contraction. The wound environment may accelerate tumor growth and subsequently lead to wound dehiscence. In addition to clarifying the clinical aspects of wounds containing tumor cells, studies of the kinetics of cellular events in such wounds would give insight into the environmental, humoral, and cellular factors stimulating tumor growth.

Two further questions then arise: does the healing process in ‘cancer-contaminated’ tissue proceed in a similar way as in the healthy tissue, and does the healing process stimulate proliferation of individual tumor cells present in the wound? Even though the mechanical dissemination of cancer cells during surgery has been discussed in the medical literature since the end of the 19th century, and was extensively studied in the 1960s, the literature on the cellular events occurring in healing cancer wounds is sparse and inconsistent [20-22]. A few studies showed correlations between events occurring during wound healing and the processes of cancer spread; however, they did not answer the basic issue of the effect of the wound environment on tumor cell growth. In order to address this issue, a comparison of wound healing of normal and cancer-contaminated tissues is required.

The aim of the current study was to compare, using immunohistochemical methods, the healing of normal intestinal anastomoses and anastomoses performed in rats with disseminated cancer, that is, the CC531 rat colon cancer model.

## Methods

### Animals

We used male Wistar AG (WAG) rats (250 to 300 g body weight; 8 to 9 weeks old), bred and maintained in our own facility. Rats were maintained in standard conditions, and received rodent laboratory chow and water *ad libitum*.

All experimental animals were treated in accordance with the guidelines of the ethics commission of the Polish Academy of Science.

### CC531 cancer cells

CC531 is a moderately differentiated and weakly immunogenic adenocarcinoma of the colon which is induced by 1,2-dimetylhydrazine and is syngenic to WAG/Rij rats. CC531 cells (kindly provided by Dr P Kuppen, Leiden University Medical Centre, the Netherlands) were cultured in RPMI medium supplemented with 10% FCS, penicillin, streptomycin, fungizon (all from Gibco, Breda, the Netherlands) and ceftriaxone (Polpharma, Warsaw, Poland). The cultures were maintained in plastic tissue culture flasks, and incubated in 5% CO_2_ at 37°C in a humidified incubator. Tumor cells were harvested from sub-confluent cultures (80 to 90% confluence) by brief (10 minutes) exposure to trypsin (Gibco) diluted 1:10 in PBS without Ca^2+^ or Mg^2+^ (Gibco). Cells were suspended in PBS without Ca^2+^ or Mg^2+^ supplemented with 10% FCS, and centrifuged at 400 g for 10 minutes, then resuspended in serum-free PBS without Ca^2+^ or Mg^2+^, and centrifuged as before. Cell viability was determined by the Trypan blue exclusion method, and was always greater than 90% [23,24].

### Inoculation of CC531 cells

Rats (n = 20) were anesthetized with ether. A mid-line incision 2 cm long was made in the abdominal wall. A suspension of 2 × 10^6^ CC531 cells in 0.5 ml 0.9% NaCl was prepared for each animal, and injected into the portal vein. Abdominal wounds were sutured. Liver and peritoneal metastatic-type tumors developed 4 to 6 weeks after CC531 inoculation. Out of the 20 rats, 4 did not develop any metastasis, 2 had individual tumor foci in the liver, and 2 had advanced cancer with peritonitis. These 8 out of 20 rats that did not match the study criteria were excluded from the study and euthanized by decapitation.

For the study, we used a homogeneous group of 12 rats with at least four tumor metastases 4 mm in size to the liver, and a few individual peritoneal tumors but no peritonitis. Tumor stage was assessed during the intestinal resection.

### Intestinal anastomoses

We performed excisions of a short segment (approximately 10 mm long) of the distal intestine with subsequent end-to-end restoration of intestinal continuity in two groups of rats. Group 1 consisted of 12 rats with CC531 colon cancer metastatic tumors, while group 2 (the control group) consisted of 12 normal healthy rats.

### Bromdeoxyuridine administration

Six rats were randomly chosen from both the cancer and control groups. Intraperitoneal injection of bromodeoxyuridine (BrdU) 10 mg was administered daily to each rat on days from 1 to 3 during the observation period.

### Immunohistochemistry of intestinal wounds

On days 3, 7 and 14, samples of anastomotic wounds were taken. Samples were cut on a cryostat into sections 5 μm thick, which were mounted onto polylysine-treated slides. Cryosections were fixed in alcohol for hematoxylin and eosin and for trichrome staining. For immunohistochemical staining, cryosections were fixed in cold acetone for 10 minutes, then air-dried, and incubated with goat serum (diluted 1:1 in Tris-buffered saline) for 20 minutes, followed by incubation for 30 minutes with primary mouse monoclonal antibodies against OX6 (for major histocompatibility complex class II; MHC II), ED1 (rat monocytes and macrophages), W3/13 (leucocytes), HIS52 (vascular endothelium), and BrdU (proliferating cells) (all from Serotec, Oxford, UK) and anti-CC531 (kindly provided by Dr P Kuppen as before). The specificity of immunostaining was confirmed by incubation of some sections without primary antibody. The antibody reactions were visualized using the LSAB-2 Alkaline Phosphatase Kit (Dako, Glostrup, Danmark), in accordance with the manufacturer’s instructions.

The cell subpopulations infiltrating the wound site were counted in five microscopic areas (×400 magnification) of intestinal wounds of normal and cancer-bearing rats using light microscopy with microimage software (Olympus, Japan).

Blood vessels in the wound were counted in five microscopic areas (×200 magnification) as the number of vessels per field; and the result was expressed as a semi-quantitative scale: +, 0 to 1 vessel/field; ++, 2 to 5 vessels/field; +++, 6 or more vessels/field. Identification of vessels was achieved using the method specified by Weidner for blood vessels counts: any stained endothelial cell or cell cluster separated from another microvessel structure was considered a countable microvessel [25].

Staining with the anti-CC531 antibody allowed counting if the number of individual tumor cells implanted into the intestinal anastomosis. Counting was performed in five microscopic fields (×400 magnification) using light microscopy with microimage software (Olympus, Japan), and results were expressed as a semi quantitative scale: +, 0 to 5 cells/field; ++, 6 to 10 cells/field; +++, 11 or more cells/field.

The population of BrdU-positive cells was divided into mononuclear infiltrating cells and CC531 cells the latter were recognized by their large, irregular shape. Both populations were counted in five microscopic fields (×400 magnification) using light microscopy with microimage software (Olympus, Japan), and results were expressed as a semi-quantitative scale: +, 0 to 5 cells/field; ++, 6 to 10 cells/field; +++, 11 or more cells/field.

Deposition of collagen in trichrome stained specimens was estimated by measuring the thickness of the blue-stained collagen bundles in the section using light microscopy with microimage software (Olympus, Japan), expressed as a semi-quantitative scale: +, 2 μm; ++, 4 μm; +++, 6 μm.

The slides were reviewed independently by three observers (WLO, MS, and MG). In the event of discrepancies between observers, the slides were reviewed once again, and results agreed upon by consensus.

### Statistics

Results are presented as percentages (mean ± SD). For statistical analysis the nonparametric Wilcoxon’s rank sum test and *t*-test were used. *P* < 0.05 was considered significant.

## Results

### Dehiscence of intestinal anastomosis

In the group of rats with cancer metastases (group 1), the dehiscence ratio was 50%. There were six cases of anastomotic dehiscence in total: one each on post-operative days 2 and 3, and two each on days 5 and 7. In the group of 12 normal rats (group 2), the dehiscence ratio was 16.6%, with two cases of anastomotic dehiscence in total: one each on post-operative day 2 and 3.

### Mononuclear infiltrates of intestinal anastomoses in cancer-bearing versus normal rats

**Figure 1:**
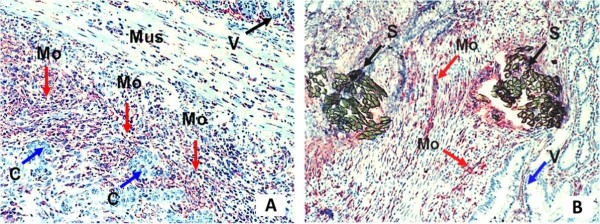
**Intestinal anastomosis performed in cancer and normal tissue. (A)** Cancer-colonized tissue, and **(B)** normal intestinal tissue, stained with anti-ED1-specific antibody for macrophages. Original magnification × 100. C, cancer islands; Mo, mononuclear cells (macrophages); Mus, muscularis mucosa; S, Suture; V, villi.

The mean number per field (×400 magnification) of mononuclear cells infiltrating the anastomosis in cancer-bearing versus normal rats, respectively, was as follows: CD14 cells 33.91 ± 7.58 versus 36.20 ±7.43, MHC II-positive cells 24.31 ± 7.10 versus 32.25 ± 8.05, and CD3 cells 24.74 ± 6.60 versus 35.84 ± 8.40. There were no significant differences between the two groups in the number of cells per microscopic field or the phenotypes of mononuclear cells infiltrating the intestinal anastomoses (Figure 1A,B).

### Blood vessels

**Figure 2:**
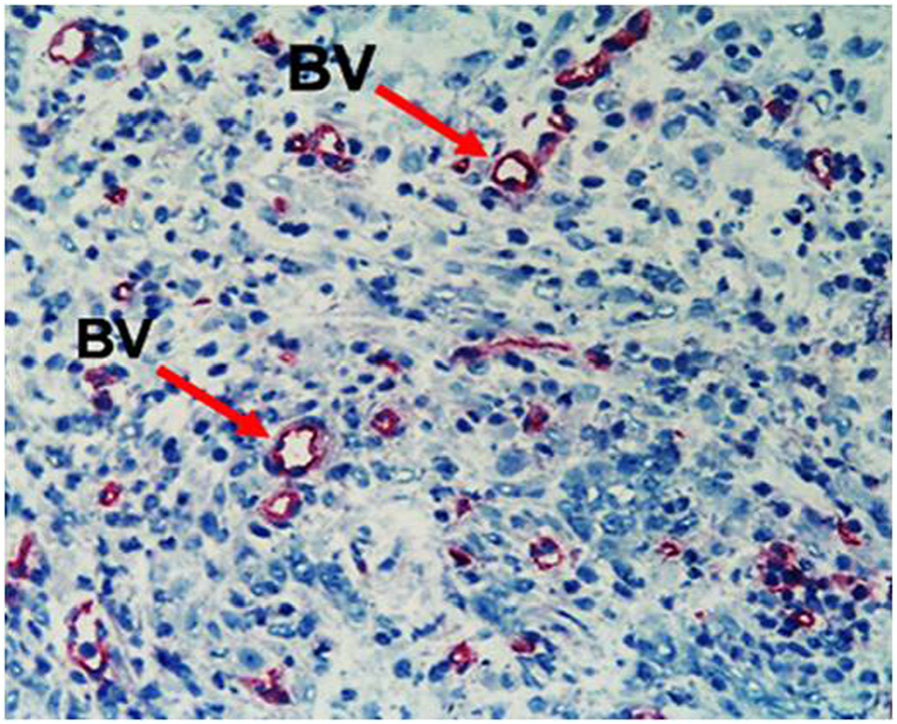
**Newly formed blood vessels in anastomotic wound in cancer-bearing rat.** Similar density of blood vessels was seen in the normal anastomotic wound. Staining with HIS 52 antibody. Original magnification × 200. BV, blood vessel.

In the intestinal anastomotic wounds of cancer-bearing rats, there were more blood vessels than in normal rats (17.33 ± 16.48 versus 13.27 ± 11.44, respectively), but this difference was not statistically significant (*P* > 0.05). Blood vessels were counted on slides obtained on post-operative day 7 and 14 from both normal and cancer-bearing rats (Figure 2).

### BrdU incorporation

**Figure 3:**
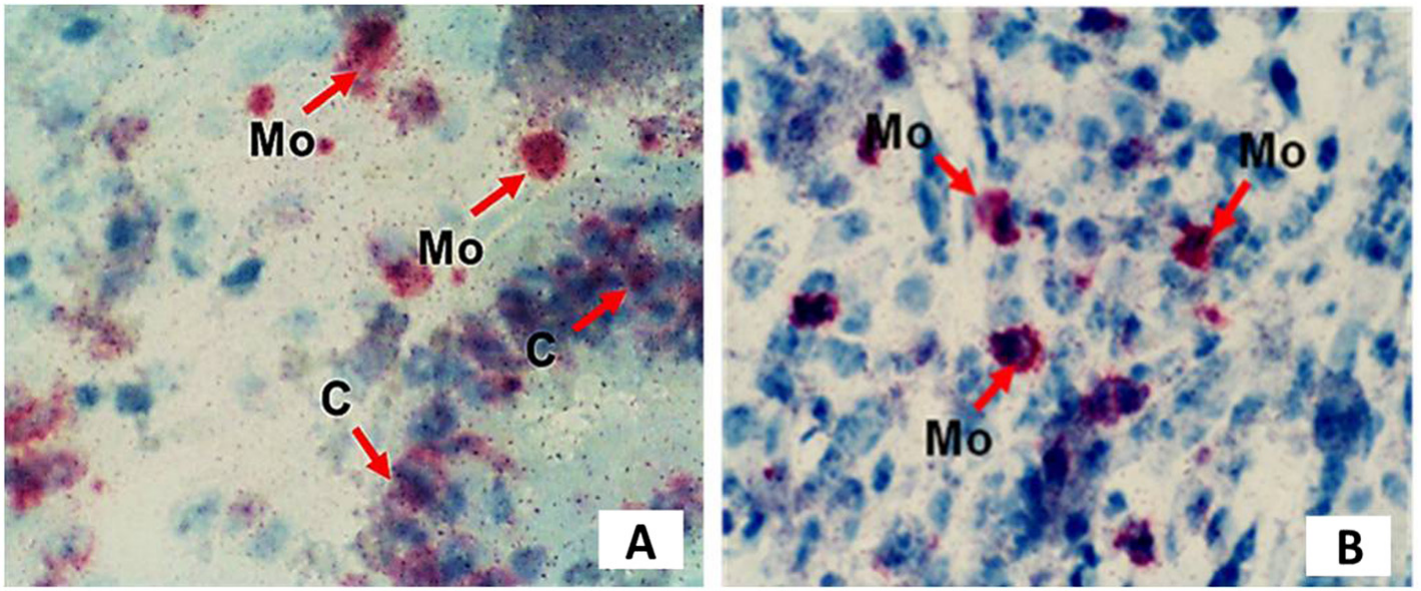
**Cancer and mononuclear cells stained for BrdU incorporation. (A)** Cancer colonized and **(B)** normal anastomosis. Original magnification × 200. C, cancer cells; Mo, mononuclear cells.

There were 18.20 ± 5.39 versus 16.44 ± 4.74 cells/field in the anastomotic wounds of the cancer-bearing and control rats, respectively (Figure 3A,B), and this difference was statistically nonsignificant (*P* > 0.05). However, staining of cancer cells for BrdU incorporation showed that there was a high density of these cells both close to and at a distance from the suture line. The area occupied by the CC531 cells reached 5 to 7% of each microscopic field. However, exact quantitative evaluation was hampered by the uneven focal distribution of the cells and their tendency to form glandular structures (Figure 3A).

### Connective tissue formation

**Figure 4:**
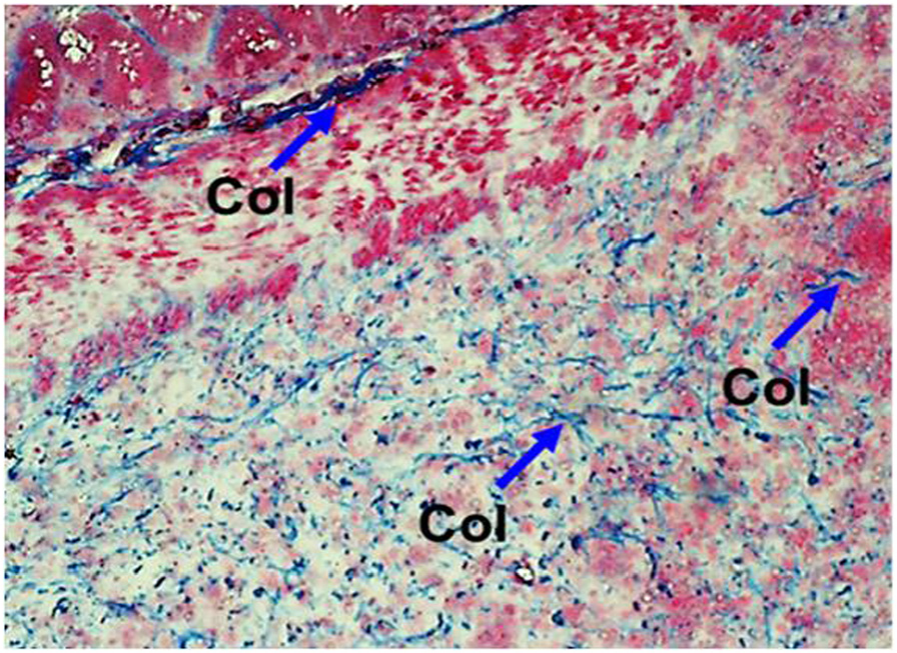
**Connective tissue formation in the intestinal anastomosis.** The cancer-bearing and normal healing tissues showed similar features. Trichrome staining, original magnification × 200. Col, collagen bundles.

We did not observe any differences in connective tissue formation or collagen deposition in the intestinal anastomotic wounds of cancer-bearing versus normal rats (Figure 4).

### CC531 implantation

**Figure 5:**
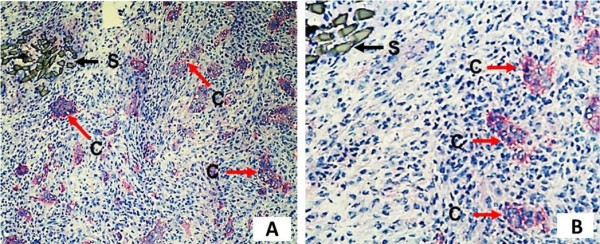
**Seeding of cancer cells forming clusters in the proximity of the anastomotic wound.** Cancer cells are stained red. Original magnification **(A)** × 100; **(B)** × 200. C, cancer cells; S, suture.

In 12 out of 12 cancer-bearing rats, CC531 cancer cells were found in the intestinal anastomotic wound, both at the edge and the central part of the healing wound. The cancer cells in the anastomotic wounds formed clusters (Figure 5A,B).

## Discussion

In the current study we observed two main groups of findings. First, there was no difference between the cancer-bearing and normal rats in 1) the number or phenotype of wound-infiltrating mononuclear cells (mainly macrophages); 2) the proliferative potential of these wound-infiltrating mononuclear cells; 3) the connective tissue formation and collagen deposition within the intestinal anastomotic wounds; or 4) the neovascularization of these wounds. Second, we found that: 1) there was multicenter seeding of cancer cells at the site of the anastomoses; 2) cancer cells formed clusters, showing their ability to proliferate; and 3) BrdU incorporation confirmed the high proliferative potential of these cells in the cancer-bearing group.

Surgical wounding may provide a favorable conditions for tumor recurrence at the site of anastomosis or in the abdominal wall [26,27]. The mechanical dissemination of cancer cells during surgery has been discussed in the medical literature since the end of the 19th century, and was extensively studied in the 1960s in animal cancer models [28]. Attention to this issue was revived by a number of authors with regard to local recurrences following anterior resections of colon and rectal cancers. In the majority of instances, the recurrences occurred in the anastomotic suture line [29-31]. Currently, wound ‘contamination’ by cancer cells at the time of operation is one of the recognized causes of local recurrence. It has been suggested that deposition of cancer cells, which may be desquamating from the tumor surface, persistent in the peritoneal fluid, or present in circulating blood and transected lymphatics, may also contribute to some recurrences [20]. In our study, we found implantation of CC531 cells in the anastomotic wounds of all the cancer-bearing rats. There may be two mechanism behind this observation: 1) cancer cells passing through the wound in the bloodstream may become mechanically trapped in the wound, and 2) the high cytokine concentration associated with wound healing may attract cancer cells in other locations to migrate to the wound and may stimulate cancer seeding and growth. Robinson and Hoppe showed that V2 rabbit carcinoma injected into the aorta implanted more frequently in limbs subjected to ischemia or blunt trauma than in normal limbs [21]. However, this tendency was not confirmed in the study by Vernick *et al*., where limbs were affected by sharp incisional trauma [22].

Disruption of normal tissue structure during tumor growth activates the host response in a manner similar to that observed during the normal wound-healing process described above. Macrophages possess a multitudinous inventory of functions, and are often described as the ‘Swiss Army knife’ of the immune system. They are recruited through the local expression of chemoattractants such as macrophage colony stimulating factor-1, monocyte chemotactic protein-1, granulocyte/macrophage colony stimulating factor, macrophage inflammatory protein-1-α, and macrophage migration inhibitory factor [32-35]. Macrophages isolated from different anatomical sites showed functional and phenotypic differences [36]. Such differences probably result from the influence of the microenvironment as well as the appropriate activation and nature of the differentiation stimulus [37,38].

Tumor-associated macrophages (TAMs) are capable of influencing a number of processes, such as matrix remodeling, angiogenesis, and stimulation of tumor growth and motility, through synthesis of growth and chemotactic factors [39]. TAMs have potential to carry out both anti-tumor and pro-tumor activities. There is a hypothesis that tumors subvert the normal functions of TAMs in order to promote tumor growth and metastasis [8,38,40,41]. Our previous studies on the adherence of mononuclear cells infiltrating CC531 liver tumors revealed a predilection of CD14 MHC II-positive cells (that is, TAMs) for liver adenocarcinoma metastases, with the highest propensity being for adherence to tumor stroma [42]. TAMs and wound macrophages have functional similarities to one another; for instance, less cytotoxic activity than activated macrophages, and have the capacity to affect angiogenesis, stroma formation, and dissolution [43,44]. Macrophages contribute to the process of angiogenesis by releasing angiogenic factors and secreting factors that stimulate other cell types, such as fibroblasts and endothelial cells [45].

Angiogenesis is marked by endothelial cell migration and capillary formation in the proliferative healing phase. Capillaries supply nutrients for granulation and tissue deposition. Failure of this process results in lack of healing. Neovascularization plays a crucial role in successful wound healing, and is probably regulated by FGF-2 and VEGF [46]. Fibroblasts play a crucial role in scar tissue formation during the proliferation, repair, and remodeling phases, but also support the process of stroma formation during tumor growth. Fibroblasts produce a number of growth factors (including FGF, EGF, PDGF, and TGF-β), and ECM components (such as collagen, elastin and proteoglycans), which serve in wound and tumor stroma formation. Fibroblasts also produce matrix metalloproteinases and TIMPs, which play crucial roles in remodeling and repair of scar tissue and tumor stroma. It has been shown that interactions between tumor cells and normal fibroblasts enhance the invasive and metastatic potential of the tumor cells [47-49].

Although the presence of cancer at the anastomotic margin is rarely encountered in modern-day treatment, as the principles of negative proximal and distal margins are well appreciated, it is possible that if cancer cells are present in the distance, they may migrate into the healing wound, and cause local recurrence. Our study on intestinal anastomosis focused on the clinical situation encountered in patients with disseminated cancer and nodal and hepatic metastases, after intestinal anastomoses performed with apparently ‘clean’ margins. In the current study, we have a higher dehiscence rate of intestinal anastomoses in the group of cancer-bearing rats versus normal rats (50% versus 16.6%, respectively). The early dehiscence rate on days 2 and 3 were equal in both groups, and were probably associated with technical errors, whereas the late dehiscence in the cancer-bearing group was probably associated with tumor proliferation and facilitated by the mechanisms described above. The most important finding was that the dehiscence rate did not correlate significantly with the impairment of the healing process seen in the histologic examinations. There were no significant differences between the normal and cancer-bearing rats in either the number or the phenotypes of the wound-infiltrating cells. In both groups, BrdU incorporation showed similar proliferative potential for the wound-infiltrating cells. There were also no significant differences between the groups in blood vessel formation or collagen deposition. Histology did not reveal any differences in the wound-formation process with the exception of the multicenter seeding of cancer cells at the site of anastomosis in the cancer-bearing group. In the intestinal anastomotic wounds, the cancer cells formed clusters, showing their ability for proliferation. Additionally BrdU incorporation confirmed the high proliferative potential of these cells. Similarly to other reports (Paget’s ‘seed and soil’ hypothesis) [50], we observed preferential growth of tumor cells in the healing wounds. We hypothesize that the high cytokine concentrations generated by the local healing process may attract cancer cells from distant tissues to migrate to and proliferate within the wound, and that excessive production of connective tissue forms a permissive microenvironment for the growth of colon carcinoma cells. The growing tumor cells will then stretch the wound, hampering the process of its contraction and causing anastomotic dehiscence.

## Conclusions

Our study particularly applies to the clinical situation encountered during palliative operations in patients with disseminated colon cancer. In addition to the technical contraindications for anastomosis or colostomy in such patients, the high probability of anastomotic dehiscence due to cancer wound seeding as shown in our study should be considered when deciding whether to perform anastomosis or colostomy in these patients.

## Abbreviations

BrdU: Bromodeoxyuridine; ECM: Extracellular matrix; EGF: Epidermal growth factor; FCS: Fetal calf serum; PBS: Phosphate-buffered saline; MHC: Major histocompatibility complex; PDGF: Platelet-derived growth factor; TAMs: Tumor-associated macrophages; TGF: Transforming growth factor; TIMPs: Tissue inhibitors of metalloproteinases; VEGF: Vascular endothelial growth factor.

## Competing interests

The authors declare that they have no competing interests.

## Authors’ contributions

MS performed experiments and histological evaluation, and wrote the manuscript. WLO supervised experiments, evaluated the histology, and corrected the manuscript. MG performed experiments and staining, evaluated the histology, and corrected the manuscript. MM consulted and corrected the obtained data and the manuscript. All authors read and approved the final manuscript.
